# Growth differentiation factor-15 levels and the risk of contrast induced acute kidney injury in acute myocardial infarction patients treated invasively: A propensity-score match analysis

**DOI:** 10.1371/journal.pone.0194152

**Published:** 2018-03-12

**Authors:** Ling Sun, Xuejun Zhou, Jianguang Jiang, Xuan Zang, Xin Chen, Haiyan Li, Haitao Cao, Qingjie Wang

**Affiliations:** Department of Cardiology, Changzhou No.2 people’s Hospital, Affiliated to Nanjing Medical University, Changzhou, China; Universita degli Studi di Perugia, ITALY

## Abstract

**Background:**

Growth differentiation factor-15 (GDF-15) is an emerging biomarker for risk stratification in cardiovascular disease. Contrast-induced acute kidney injury (AKI) is an important complication in patients undergoing coronary angiography (CAG) or percutaneous coronary intervention (PCI). In this retrospectively observational study, we aimed to determine the role of GDF-15 and the risk of AKI in acute myocardial infarction (AMI) patients.

**Methods:**

The medical records of 1195 patients with AMI were reviewed. After exclusion criteria, a total of 751 eligible patients who underwent CAG or PCI were studied. Preoperative clinical parameters including GDF-15 levels were recorded. Multivariate logistic regression analysis was used to identify the risk factors of AKI. Subsequently, to reduce a potential selection bias and to balance differences between the two groups, a propensity score-matched analysis was performed. We recorded the 30-day all-cause mortality of the total study population. Kaplan-Meier analysis was performed to identify the association between short term survival in AMI patients and GDF-15 level.

**Results:**

Among 751 enrolled patients, 106 patients (14.1%) developed AKI. Patients were divided into two groups: AKI group (n = 106) and non-AKI group (n = 645). GDF-15 levels were significantly higher in AKI group compared to non-AKI group (1328.2 ± 349.7 ng/L vs. 1113.0 ± 371.3 ng/L, P <0.001). Multivariate logistic regression analyses showed GDF-15 was an independent risk factor of AKI (per 1000 ng/L increase of GDF-15, OR: 3.740, 95% CI: 1.940–7.207, P < 0.001). According to GDF-15 tertiles, patients were divided into three groups. Patients in middle (OR 2.93, 95% CI: 1.46–5.89, P = 0.003) and highest GDF-15 tertile (OR 3.72, 95% CI: 1.87–7.39, P <0.001) had higher risk of AKI compared to patients in the lowest GDF-15 tertile. The propensity score-matched group set comprised of 212 patients. Multivariate logistic regression revealed that GDF-15 is still an independent risk factor for AKI after matching (per 1000 ng/L increase of GDF-15, OR: 2.395, 95% CI: 1.020–5.626, P = 0.045). Based on the Kaplan-Meier analysis, the risk of 30-day all-cause mortality increased in higher GDF-15 tertiles log rank chi-square: 29.895, P <0.001).

**Conclusion:**

This suggests that preoperative plasma GDF-15 is an independent risk factor of AKI in AMI patients underwent CAG or PCI. GDF-15 and AKI are associated with poor short term survival of AMI patients.

## Introduction

Contrast induced acute kidney injury (AKI) is a significant and common complication in patients with acute myocardial infraction (AMI) undergoing coronary angiography (CAG) or percutaneous coronary intervention (PCI). An increasing number of studies have shown that AKI is associated with increased mortality and morbidity in AMI patients[1–3]. However, the pathophysiology of contrast induced AKI remains unclear. There are polyfactorial pathophysiologies of this complication, including inflammation and oxidative stress, hemodynamic disorders, and renal ischemia-reperfusion injury. There is no completely successful prevention for this disease[4, 5]. Thus, identification of AMI patients who may be able to develop AKI after interventions is necessary to prevent AKI in the future[6, 7].

Some risk biomarkers are thought to be related to AKI. Different scores have been developed based on these risk factors to predict the incidence of AKI after CAG or PCI[2, 8, 9]. For example, AGEF risk score, a 3-variables clinical risk score based on age, left ventricular ejection fraction (LVEF), and estimated glomerular filtration rate (eGFR), was able to predict contrast-induced nephropathy (CIN) in AMI patients undergoing PCI[10]. However, all risk scores exhibited low predictive accuracy for AKI and 3-year MACEs[11]. Thus, it is necessary to find a biomarker for AKI which could be easily measured before CAG or AKI and is more accurate in predicting AKI.

Some potential candidates might be the cardiac or renal biomarkers. In addition, the plasma concentration of the Growth Differentiation Factor-15 (GDF-15) is affected both by cardiac and kidney dysfunction. Studies have shown that GDF-15 is a predictive biomarker for no-reflow, short-term and long-term outcome after AMI[12–14]. Preoperative plasma concentration of GDF-15 is an independent predictor of morbidity, short-term and long-term mortality, in patients undergoing cardiac surgery[15]. GDF-15 was also found to be a predictor for AKI in patients undergoing cardiac surgery[16].

In this study, we hypothesized that the pathophysiology of contrast induced AKI and reperfusion injury in AMI patients undergoing CAG or PCI, could be similar related to GDF-15. Therefore, the aim of this study was to explore the relationship between AKI and GDF-15, in order to determine the high risk of AKI patients and improve the prognosis.

## Methods

### Ethical statements

The study was conducted in Changzhou No.2 People’s Hospital in Jiangsu, China. This study protocol was approved by Changzhou No.2 People’s Hospital ethics committee on August 2017 and the study was performed in accordance with the Declaration of Helsinki. We obtained written informed consent from all patients (except death) before the use of their medical record data.

### Study populations

**Fig 1:**
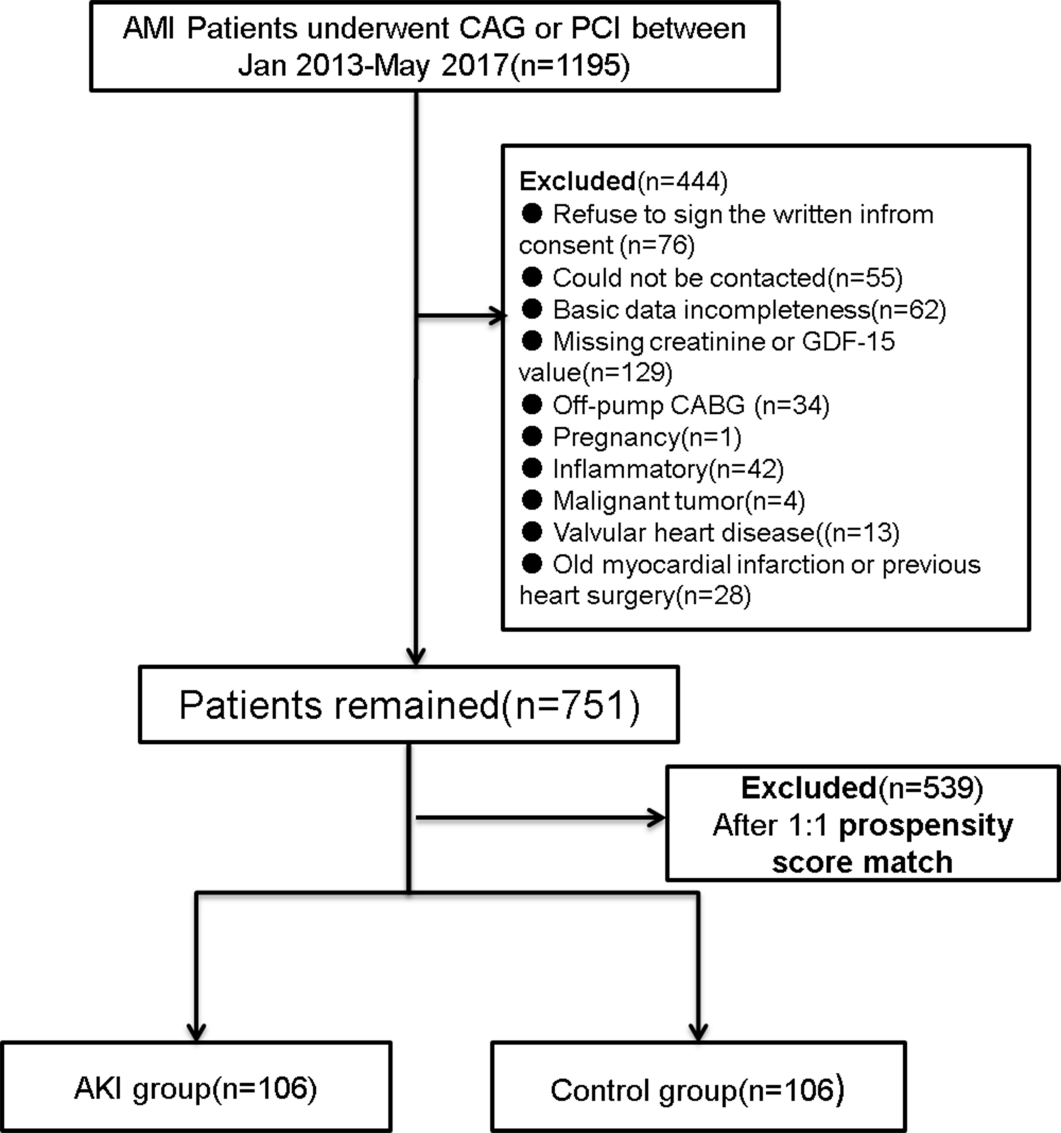
Study flow chart. AMI = acute myocardial infraction; CAG = coronary angiography; PCI = percutaneous coronary intervention; eGFR = estimated glomerular filtration rate; AKI = acute kidney injury.

Between March 2013 and May 2017, we retrospectively reviewed the electronic medical records of inpatients diagnosed with AMI in department of cardiology. The inclusion criteria included the followings: all eligible patients were more than twenty years old. The definition of AMI was according to the third universal definition of myocardial infarction from the Joint ESC/ACCF/AHA/WHF Task Force[17]. All enrolled patients received CAG or PCI therapy after admission. Exclusion criteria included the followings: could not be contacted (n = 55), refused to sign the written inform consent (n = 76), basic data incompleteness (n = 62), missed creatinine or GDF-15 value (n = 129), off-pump CABG (n = 34), pregnancy (n = 1), valvular heart disease (n = 13), old myocardial infarction or previous heart surgery(n = 28), inflammatory (n = 42), malignant tumor(n = 4), trauma (n = 0), autoimmune disease (n = 0), severe hepatic dysfunction (n = 0), malignant anemia (n = 0), severe sepsis (n = 0). (Fig 1)

### Clinical variables collection

All the baseline clinical data were collected from the medical records. The following clinical variables were recorded: age, gender, systolic blood pressure (SBP), diastolic blood pressure (DBP), body mass index (BMI), heart rate, smoking, alcohol intake, diabetes mellitus, hypertension, laboratory findings, and medications history. The procedural characteristics were also recorded: contrast exposure time, contrast volume, number of stents, the use of isotonic contrast agent, and whether receiving a hydration treatment.

### Laboratory parameters

Blood samples of the enrolled patients were collected and analyzed immediately on admission and on the second day morning as the baseline level. The following measurements were made: white blood cell count (WBC), neutrophil ratio, hemoglobin, cardiac troponin I (cTnI), serum creatinine, uric acid, albumin, total cholesterol (TC), high-density lipoprotein cholesterol (HDL-C), low-density lipoprotein cholesterol (LDL-C), heamoglobinA1c (HbA1c). Serum creatinine was measured again within 48 hours after intervention. All biochemical measurements were performed using standard laboratory techniques. All analyses were performed by investigators blinded to the clinical data of the patients.

### GDF-15

Blood samples for determination of GDF-15 were taken preoperatively (before CAG or PCI). Blood samples were separated and stored at -80 C until measurement. Analyses were accomplished within 48 hours. GDF-15 was measured by enzyme-linked immunosorbent assay (ELISA) with a limit of detection of 4.39 pg/ml and a linear range from 23.4–1500 pg/ml (Quantikine, R&D Systems, USA). The color intensity, relative to GDF-15 concentrations, was measured at 450 nm with a spectrophotometer (BioTek, Winooski, VT, United States).

### Definition of AKI and calculating of eGFR

Serum creatinine was measured one day before CAG or PCI as baseline level. According to the Kidney Disease Improving Global Outcomes (KDIGO) criteria[18]. AKI was defined as “an absolute increase of serum creatinine of more than or equal to 0.3mg/dL or increase to more than or equal to 150% from baseline within any 48 hour during hospital days”. According to the occurrence of AKI development, patients were divided into 2 groups: AKI group and non-AKI group.

The eGFR was calculated by the abbreviated MDRD equation according to the baseline serum creatinine concentration[19].

### Outcome study

The primary outcome was the development of AKI after procedure. The secondary outcome of the study was 30 days of all-cause mortality. The 30 days mortality was recorded in the electronic medical record system by telephone contacts from trained nurses.

### Statistical analyses

The research data is collected on a standard form to check for completeness. All data were doubled keyed into a database. SPSS software (version 22.0, IBM Corp. Armonk, NY, USA) was used for statistical analyses. Missing data other than serum creatinine concentration or GDF-15 were presented in less than 2% of the record. We validated the sample size to a rule that the number of outcome events should be ten per every independent risk factor[20]. In our study, the sample size was calculated to be 500 patients or more to allow unbiased accommodation of less than ten predictors in a multivariable logistic regression analysis under the assumption of at least 20% incidence of AKI. A propensity score matching was estimated to reduce potential selection bias and to balance differences between AKI group and non-AKI group. Patients were matched using a greedy method with 1:1 pair. A total of 106 patients in AKI group were matched with 106 patients in non-AKI group. Variables used as contributors to the propensity score included the following: age, gender, hypertension, diabetes mellitus, alcohol intake, WBC, neutrophil ratio, HDL-C, HbA1c, contrast volume > 150 ml, contrast exposure time > 60 min, the use of isotonic contrast agent, whether receiving PCI, and whether receiving a hydration treatment. The caliper was defined as 0.15 standard deviation of the logit transformed propensity score. Multivariable logistic regression analysis was performed in the matched sample again. The balance between the two groups was analyzed using Paired comparison of contributor variables.

Continuous variables were described using mean ± standard deviation or median, and 25^th^ and 75^th^ percentiles. Categorical variables were expressed by absolute values (percentages). Comparisons of differences between two groups were performed using Student t test for continuous variables and chi-square or Fisher exact test for categorical variables. Multivariate logistic regression analysis was performed to identify whether GDF-15 was an independent risk factor of AKI. The adjusted odds ratio (OR) and 95% confidence interval (CI) were calculated. The prediction value of GDF-15, serum creatinine and multivariate logistic regression model were determined by the area under the receiver operating characteristics (ROC) curves. Kaplan Meier analysis was used to show the association of baseline GDF-15 values and 30-day all-cause mortality risk. Moreover, the association of AKI and 30-day all-cause mortality risk were also compared using Kaplan Meier analysis and log rank test. For all analyses, P value of less than 0.05 (2-sided significance testing) was considered statistically significant.

## Results

### Basic clinic characteristics

A total of 751 patients were analyzed following exclusion protocol explained in the methods (Fig 1). There were 106 patients (14.1%) developed AKI as defined by the KDIGO criteria. GDF-15 levels were significantly higher in AKI group compared with non-AKI group (1328.2 ± 349.7 ng/L vs. 1113.0 ± 371.3 ng/L, P < 0.001). The eGFR was significantly lower in AKI group compared to non-AKI group (50.7 ± 28.5 ml/min vs. 72.6 ± 28.9 ml/min, P < 0.001). (Table 1)

**Table 1:** Basic clinical and procedural characteristics between AKI group and non-AKI group.

Variables	AKI Group(n = 106)	Non-AKI Group(n = 645)	P-value
**Demographics**			
Age, y	73.2±14.0	65.4±13.0	<0.001
Male, n%	65(61.3%)	462(71.6%)	0.032
BMI, Kg/m^2^	22.3±4.2	23.5±9.7	0.217
SBP, mmHg	128.3±22.1	126.0±22.0	0.314
DBP, mmHg	79.7±20.6	81.2±19.1	0.484
Heart rate, bpm	84.6±19.0	82.0±15.7	0.191
Killip class≥ 3, n%	60(56.6%)	232(36.0%)	<0.001
**Medical history, n%**			
Smoking	47(44.3%)	339(87.8%)	0.117
Alcohol intake	8(7.5%)	100(15.5%)	0.03
Hypertension	84(79.2%)	391(60.6%)	<0.001
Diabetes	38(35.8%)	150(23.3%)	0.006
**Medications, n%**			
ACEI/ARB	94(88.7%)	551(85.4%)	0.373
β-blocker	70(66.0%)	406(62.9%)	0.54
CCB	2(1.9%)	16(2.5%)	0.978
Diuretics	37(34.9%)	262(40.6%)	0.265
Statin	106(100%)	642(99.5%)	0.482
**Laboratory measurements**			
Glucose(mmol/L)	12.4±4.5	12.7±4.3	0.611
Serum creatinine, μmol/L	131.6±79.2	88.4±46.1	<0.001
eGFR, mL/min/1.73m^2^	50.7±28.5	72.6±28.9	<0.001
TC,mmol/L	4.18±1.07	4.23±1.02	0.675
HDL-C, mmol/L	1.25±0.31	1.15±0.35	0.003
LDL-C, mmol/L	2.36±0.71	2.47±0.83	0.154
Uric acid, μmol/L	362.0±170.3	346.9±167.3	0.399
Serum albumin, g/L	37.3±4.6	36.9±4.5	0.348
WBC, 10^9^/L	10.7±3.9	9.3±3.5	0.001
Neutrophil ratio (%)	81.2±10.0	75.0±13.2	<0.001
Anemia, n%	10(9.4%)	8(1.2%)	<0.001
HbA1c, %	7.04±1.95	6.62±1.62	0.016
GDF-15, ng/L	1328.2±349.7	1113.0±371.3	<0.001
**Procedural characteristic**			
Contrast volume, mL	85.9±55.9	101.2±56.1	0.012
Contrast exposure time, min	54.1±33.3	63.0±34.2	0.009
LAD	27(25.5%)	227(35.2%)	0.072
LCX	8(7.5%)	123(19.1%)	0.003
RCA	29(27.3%)	120(18.6%)	0.008
use of isotonic contrast agents	74(69.8%)	213(33.0%)	<0.001
hydration therapy	74(69.8%)	213(33.0%)	<0.001
PCI	64(60.4%)	462(71.6%)	0.019

AKI = acute kidney injury, BMI = body mass index, SBP = systolic blood pressure, DBP = diastolic blood pressure, ACEI/ARB = angiotensin-converting enzyme inhibitor/angiotensin receptor blocker, CCB = Calcium channel blocker, eGFR = estimated glomerular filtration rate (mL/min/1.73m^2^), TC = total cholesterol, HDL-C = High-density lipoprotein cholesterol, LDL-C = Low-density lipoprotein cholesterol, WBC = white blood cell, Anemia was defined using World Health Organization criteria: baseline hematocrit value <39% for men and <36% for women, GDF-15 = growth differentiation factor-15, HbA1c = glycated hemoglobin, LAD = left anterior desending, LCX = left circumflex, RCA = right coronary artery, PCI = percutaneous coronary intervention.

Basic clinical and procedural characteristics, preoperative medications and laboratory measurements are presented in Table 1. Compared with subjects without AKI, the patients in AKI group were older, had more women, had more patients with anemia, more often presented with hypertension, diabetes mellitus, alcohol intake, Killip class ≥ 3, had higher serum creatinine and high-density lipoprotein cholesterol (HDL-C), white blood cell (WBC), neutrophil ratio and glycated hemoglobin(HbA1c) levels on admission. There were also statistical differences between compared groups with procedural characteristics as showed in Table 1.

Table 2 shows the results of both univariable and multivariable logistic analyses of GDF-15 and AKI in the total group. It indicates that GDF-15 is an independent risk factor for AKI in AMI patients in univariable logistic regression (entire sample: OR 4.546, 95% CI: 2.599–7.951, P < 0.001). Moreover, after adjusted by age and gender or adjusted by all confounding factors listed in Table 2, GDF-15 is still an independent risk factor for AKI Model 2: OR 4.486, 95% CI: 2.524–7.973, P < 0.001; Model 3: OR 3.740, 95% CI: 1.940–7.207, P < 0.001). This indicates that the adjusted incidence of AKI increases 2.74 times for each 1000 ng/L increase of GDF-15.

**Table 2:** Logistic regression analysis for GDF-15 and AKI before matching.

	β	Wald chi-square	P Value	OR (95%CI)
GDF-15 (Model1) Per 1000 ng/L increase	1.514	28.183	<0.001	4.546(2.599–7.951)
GDF-15 (Model2) Per 1000 ng/L increase	1.501	26.160	<0.001	4.486(2.524–7.973)
GDF-15 (Model3) Per 1000 ng/L increase	1.319	15.525	<0.001	3.740(1.940–7.207)

Data are shown as (odds ratio) OR of GDF-15; Model1: unadjusted; Model2: adjusted for age, gender; Model 3: adjusted for age ≥ 70 years, gender, smoking, acohol intake, hypertension, diabetes mellitus, anemia, Killip class ≥ 3, eGFR < 60ml/min/1.73m^2^, use of isotonic contrast agents, LAD, LCX, RCA, PCI, WBC, Neutrophil ratio, serum creatinine, Uric acid, HDL-C, HbA1c ≥ 7.0%, contrast volume > 150 ml, contrast exposure time > 60 min.

**Fig 2:**
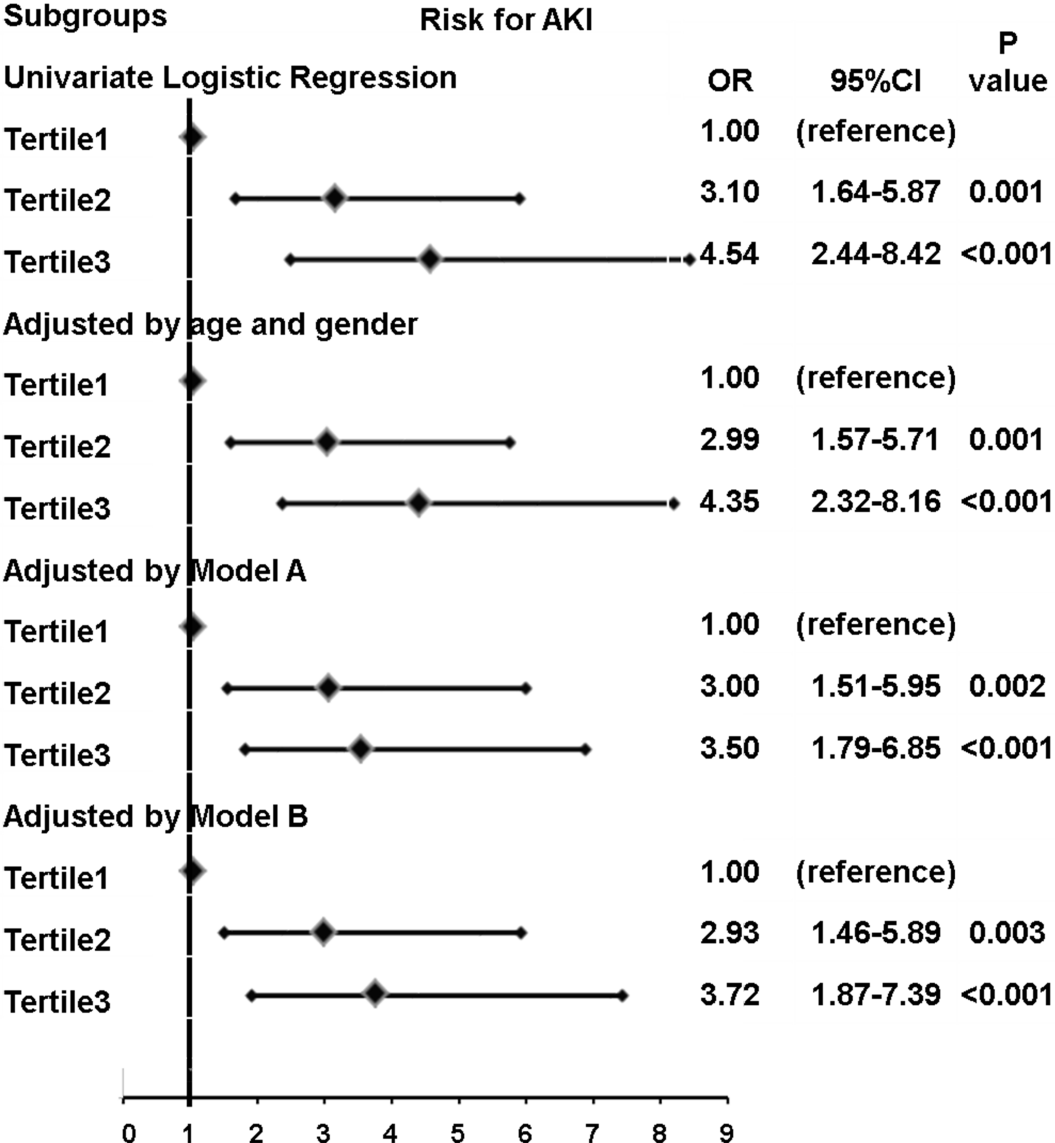
Presented are associations between GDF-15 level classified by the tertiles of the distribution and AKI. T1, the first tertile; T2, the second tertile; T3, the third tertile. Model A = logistic regression model adjusting for adjusted for age, gender, Killip class ≥ 3, diabetes mellitus, serum creatinine, WBC, Neutrophil ratio, HDL-C, anemia, HbA1c ≥ 7.0%, eGFR < 60ml/min/1.73m^2^. Model B = logistic regression model adjusting for Model A plus procedural characteristics (contrast volume > 150 ml, contrast exposure time > 60 min, LAD, LCX, RCA, PCI). T1 (GDF-15 ≤ 893.62 ng/L) T2 (893.62 < GDF-15 ≤ 1307.86 ng/L); T3 (GDF-15 > 1307.86 ng/L).

According to entire GDF-15 tertiles, patients were divided into three groups. Patients in middle and highest GDF-15 tertile had higher risk of AKI compared to patients in the lowest GDF-15 tertile after adjusted by confounding factors (Fig 2).

In our study, we also explored other predictors for AKI in logistic analyses. Other predictors included the following: age ≥ 70 years (OR: 2.008, 95%CI: 1.201–3.360, P = 0.008) serum creatinine > 132.6 μmol/L (OR: 3.210, 95%CI: 1.757–5.868, P < 0.001), anemia(OR: 5.541, 95%CI: 1.852–16.577, P = 0.002), eGFR < 60 ml/min/1.73m^2^ (OR: 1.773, 95%CI: 1.005–3.128, P = 0.048) RCA (OR: 1.766, 95%CI: 1.046–2.983, P = 0.033) hypertension (OR: 1.767, 95%CI: 1.022–3.054, P = 0.041), neutrophil ratio>76.5% (OR: 2.034, 95%CI: 1.235–3.350, P = 0.005). (Data not showed in the table).

The propensity score-matched group set comprised of 212 patients. As shown by the p-values of the t test and chi-square of Fisher exact test, the balance of both AKI and non-AKI groups was good for the parameters used for contributors to the propensity score (Table 3).

**Table 3:** Basic clinical and procedural characteristics after matching.

Variables	AKI Group(n = 106)	Non-AKI Group(n = 106)	P-value
**Demographics**			
Age, y	73.2±14.0	69.6±12.3	0.051
Male, n%	65(61.3%)	62(58.5%)	0.674
BMI, Kg/m^2^	22.3±4.2	23.2±4.0	0.099
SBP, mmHg	128.3±22.1	129.4±26.6	0.732
DBP, mmHg	79.7±20.6	77.4±18.4	0.405
Heart rate, bpm	84.6±19.0	81.4±15.6	0.189
Killip class≥ 3	60(56.6%)	54(50.9%)	0.491
**Medical history, n%**			
Smoking	47(44.3%)	43(40.6%)	0.578
Drinking,	8(7.5%)	6(5.7%)	0.58
Hypertension	84(79.2%)	83(78.3%)	0.867
Diabetes	38(35.8%)	29(27.4%)	0.184
**Medications, n%**			
ACEI/ARB	94(88.7%)	93(87.7%)	0.831
β-blocker	70(66.0%)	67(63.2%)	0.667
CCB	2(1.9%)	9(7.5%)	0.059
Diuretics	37(34.9%)	64(60.4%)	<0.001
Statin	106(100%)	104(98.1%)	0.498
**Laboratory measurements**			
Glucose(mmol/L)	12.4±4.5	12.8±4.1	0.506
Serum creatinine, μmol/L	131.6±79.2	107.2±69.9	0.019
eGFR, ml/min	50.7±28.5	60.4±31.1	0.018
TC,mmol/L	4.18±1.07	4.06±0.96	0.407
HDL-C, mmol/L	1.25±0.31	1.22±0.30	0.505
LDL-C, mmol/L	2.36±0.71	2.36±0.79	0.967
Uric acid, μmol/L	362.0±170.3	327.6±164.5	0.136
Serum albumin, g/L	37.3±4.6	37.2±4.1	0.838
WBC, 10^9^/L	10.7±3.9	10.3±3.7	0.488
N(%)	81.2±10.0	80.6±8.3	0.675
Anemia, n%	10(9.4%)	6(5.7%)	0.436
HbA1c, %	7.04±1.95	7.02±2.06	0.942
GDF-15, ng/L	1328.2±349.7	1207.4±367.1	0.015
**Procedural characteristic**			
Contrast volume, mL	85.9±55.9	86.9±57.0	0.898
Contrast exposure time, min	54.1±33.3	53.8±34.3	0.945
LAD	27(25.5%)	44(41.5%)	0.047
LCX	8(7.5%)	5(4.71%)	0.569
RCA	29(27.3%)	18(17.0%)	0.142
use of isotonic contrast agents	74(69.8%)	59(55.7%)	0.033
hydration therapy	74(69.8%)	59(55.7%)	0.033
PCI	64(60.4%)	62(58.5%)	0.493

Table 4 shows the results of both univariable and multivariable logistic analyses of GDF-15 and AKI after propensity score matching. It indicated that GDF-15 is an independent risk factor for AKI in AMI patients after adjusted by age and gender (after matching: OR 2.507, 95% CI: 1.154–5.445, P = 0.02). After adjusted by serum creatinine, eGFR, left anterior descending (LAD), use of isotonic contrast agents, and use of diuretics, GDF-15 is still an independent risk factor for AKI. The risk of AKI increased 1.395 times (after matching: OR 2.395, 95% CI: 1.020–5.626, P = 0.045) for each 1000 ng/mL increase of GDF-15.

**Table 4:** Logistic regression analysis for GDF-15 and AKI after matching.

	β	Wald chi-square	P Value	OR (95%CI)
GDF-15 (Model1) Per 1000 ng/L	0.941	5.799	0.016	2.563(1.191–5.513)
GDF-15 (Model2) Per 1000 ng/L	0.919	5.392	0.02	2.507(1.154–5.445)
GDF-15 (Model3) Per 1000 ng/L	0.873	4.018	0.045	2.395(1.020–5.626)

Data are showed as (odds ratio) OR of GDF-15; Model1: unadjusted; Model2: adjusted for age, gender; Model 3: adjusted for serum creatinine, eGFR, left anterior desending (LAD), use of isotonic contrast agents and use of diuretics.

**Fig 3:**
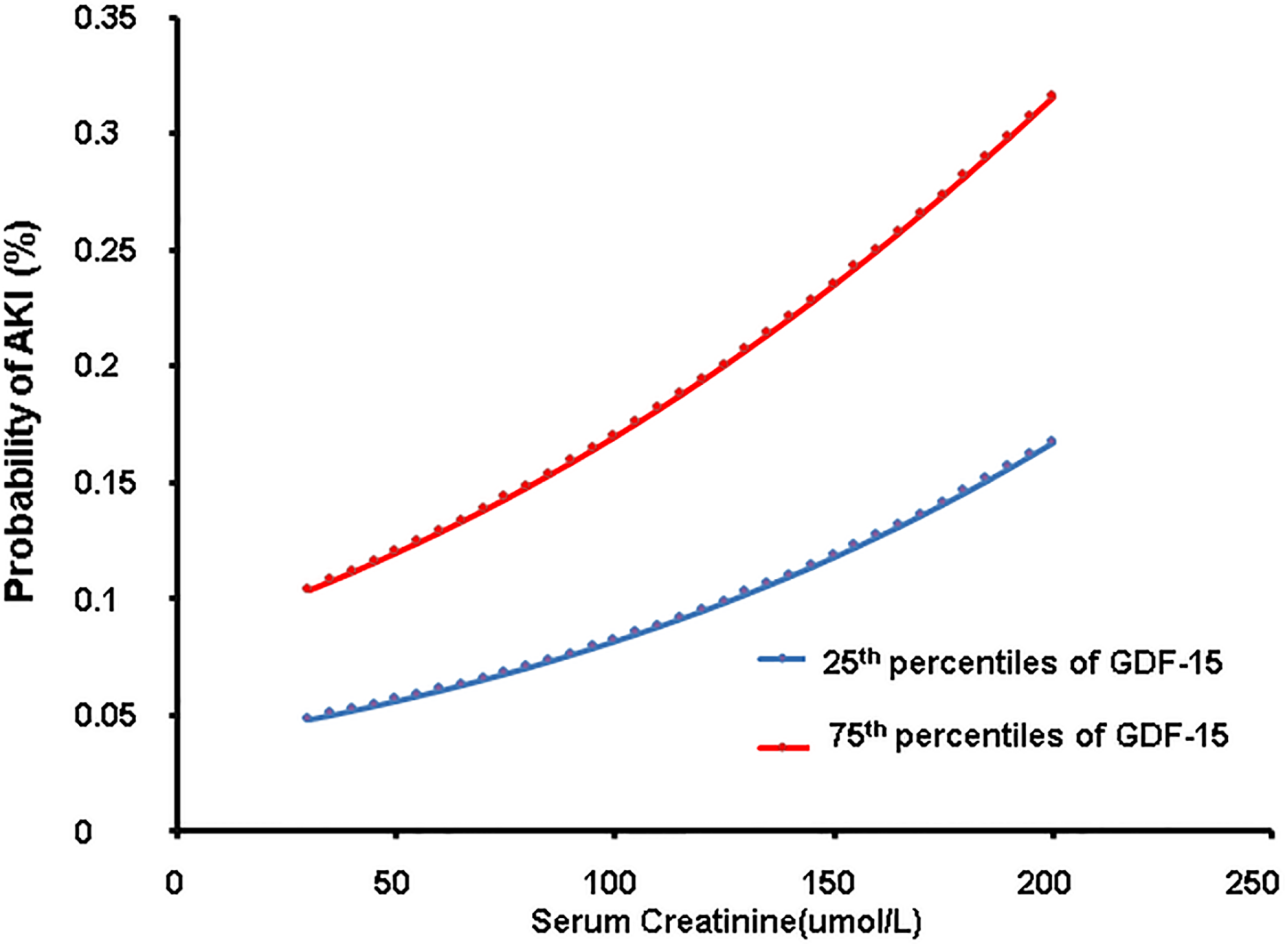
Presented are probability of AKI performed by logistic regression analyses (male, 70years). X-axis represents the concentration of serum creatinine, and Y-axis represents the probability of AKI. The blue line represents GDF-15 of 25th percentiles, while the red one represents GDF-15 of 75th percentiles.

We designed a prediction model by logistic regression analyses. As shown in Fig 3, assuming that a 70–year–old, male, there was a positive correlation between the increase in serum creatinine and GDF-15 levels and the incidence of AKI.

**Fig 4:**
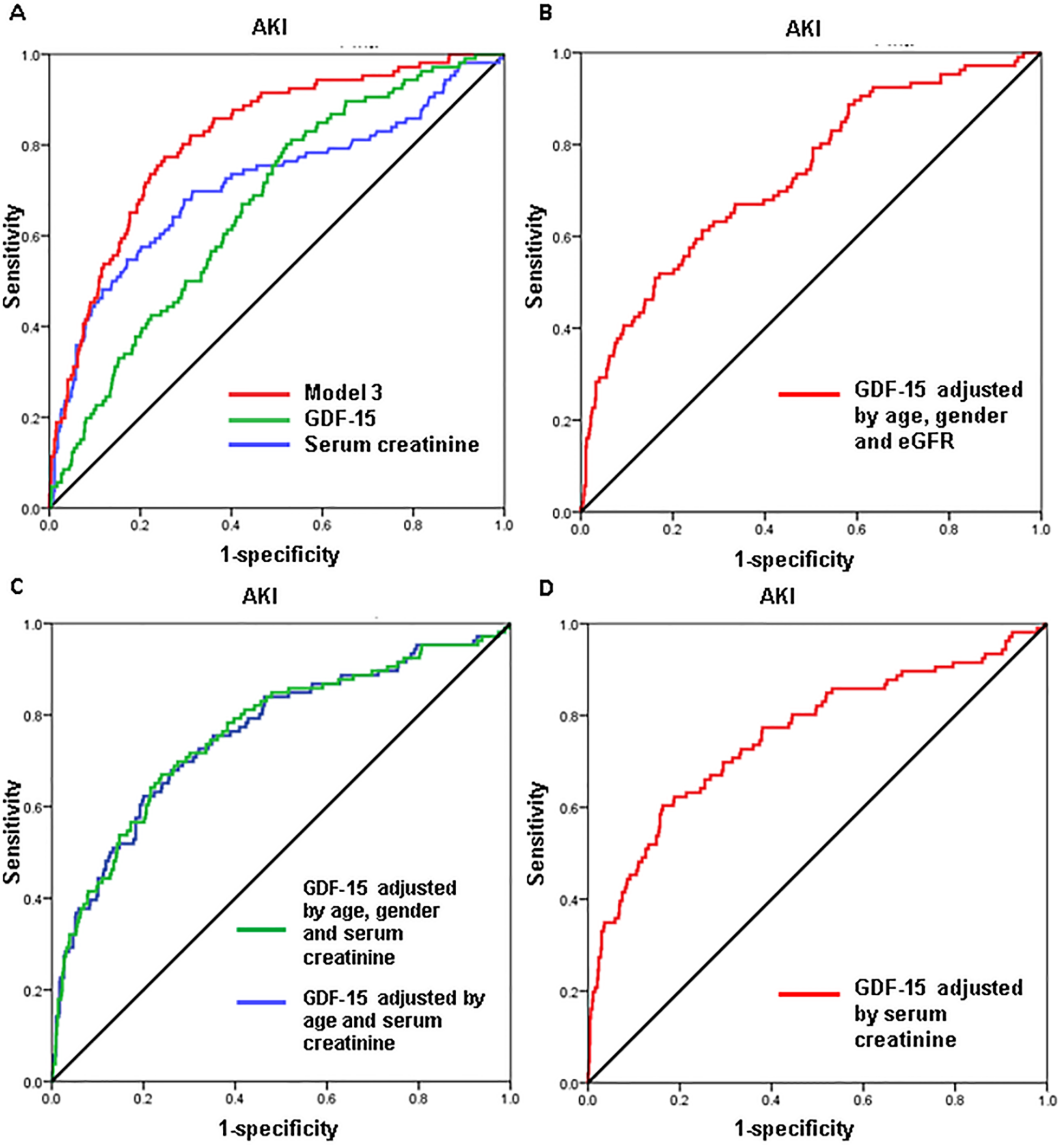
ROC analyses of biomarkers to predict AKI. AUC = area under the curve. AUC of model 3 is 0.816 (95% CI: 0.774–0.859), while AUC of GDF-15 is 0.666 (95% CI: 0.614–0.717), and AUC of creatinine is 0.714 (95% CI: 0.653–0.775). AUC of GDF-15 adjusted by age, gender and eGFR is 0.757 (95% CI: 0.701–0.813). AUC of GDF-15 adjusted by age and serum creatinine is 0.758(95% CI: 0.704–0.812), while AUC of GDF-15 adjusted by age, gender and serum creatinine is 0.761(95% CI: 0.707–0.814). AUC of GDF-15 adjusted by serum creatinine is 0.733(95% CI: 0.680–0.786).

ROC analyses showed that the AUC for GDF-15 to predict AKI was 0.666, 95% CI: 0.614–0.717, while AUC of the baseline serum creatinine was 0.714, 95% CI: 0.653–0.775. And Model 3 (which was described in Table 2) showed a better performance than GDF-15 or baseline creatinine alone (Fig 4).

**Fig 5:**
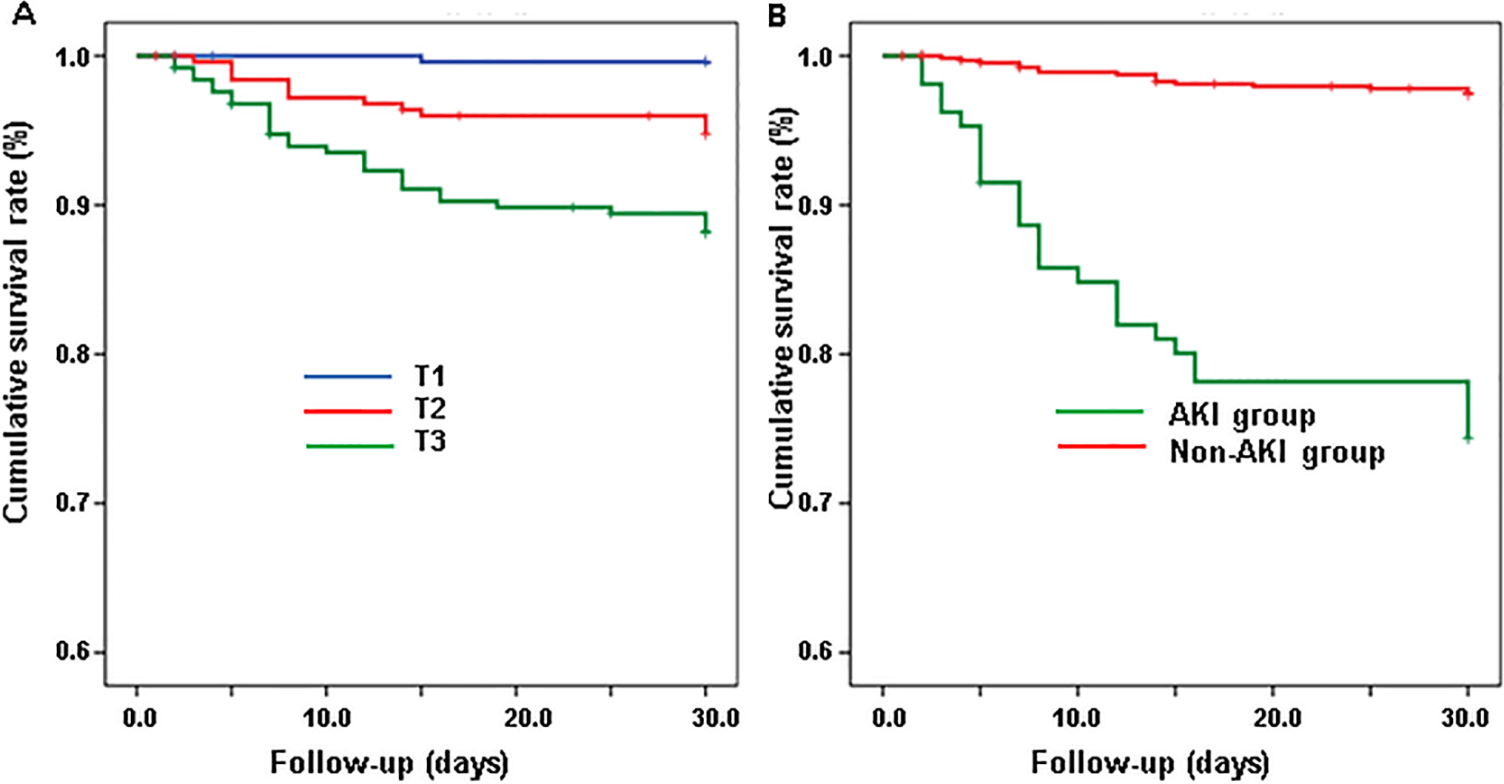
Kaplan Meier analyses of GDF-15 and AKI. Patients in higher GDF-15 tertile had higher 30-days all-cause mortality risk compared with the middle GDF-15 tertile and lowest GDF-15 tertile (log rank chi-square:29.895, P < 0.001). Patients in AKI group had higher 30-days all-cause mortality risk compared with patients in non-AKI group (log rank chi-square: 97.473, P < 0.001).

The mean follow-up of the 751 patients was 28.6 ± 5.5 days. Among these patients, 43 patients died (5.7%), including 27 in AKI group and 16 in non-AKI group. Kaplan Meier analyses shows a relevant association of baseline GDF-15 values with 30-day all-cause mortality risk using GDF-15 tertiles (Fig 5A). Furthermore, patients in AKI group had higher 30 days all-cause mortality risk compared with patients in non-AKI group in Kaplan Meier analyses. (Fig 5B)

## Discussion

Our study shows that plasma GDF-15 before the procedure is an independent risk factor of AKI in AMI patients underwent CAG or PCI. AKI is one of the most serious complications of AMI. AKI, and together with GDF-15 are also associated with poor short term survival in AMI patients.

### Prevalence

In our study, we studied 751 AMI patients in our center. The prevalence of AKI was 14.1%. In recent reports, the prevalence of AKI varied from 10–25%, and in those high risk patients, the number could be 40%[1, 21]. It indicated that there is high incidence of AKI for AMI patients in hospital, leading to increased mortality and morbidity in AMI patients[1]. It is necessary to recognize the high risk patients and prevent the development of AKI.

### GDF-15, serum creatinine and AKI

Our study shows elevated GDF-15 levels at the time of hospital admission in AMI patients is an independent risk factor for AKI in AMI patients. In our predictive probability model, the incidence of AKI increased with elevated GDF-15 levels. The propensity score match analyses showed that GDF-15 is still independent predictors of AKI after matching.

Our study shows that baseline serum creatinine is also an independent risk factor of AKI. Serum creatinine is of certain value in predicting AKI. It is reported that baseline creatinine was a strong predictor for AKI. Our study also showed a better performance to predict AKI when combined serum creatinine with GDF-15, age and gender.

### Other risk factors for AKI

Our study suggested that age, anemia, eGFR decrease, right coronary artery (RCA) lesions, high blood pressure and percentage of neutrophils were also a risk factor for AKI. It is also reported that use of diuretics[22], left anterior descending coronary artery disease[23, 24], shock and anemia were all independent risk factors[25]. Further clinical trials are needed to confirm role of these risk factors on AKI.

### Follow-ups

Short-term follow-up results indicated that the elevation of GDF-15 was associated with 30 days of mortality in patients. Patients in AKI group were of worsen prognosis.

### Limitations

This is a single-center retrospective study, although we have performed PSM methods to control confounding factors and bias, the causal relationship of GDF-15 with AKI is still uncertain. Randomized controlled study in future is requisite to explore the association with GDF–15 and AKI.

The fluctuation of GDF-15 during the patient's hospitalization was not detected in our study. The time when the blood sample collected may affect the results. The study failed to include new markers such as NGAL, CysC, KIM-1, and so on. We did not compare GDF-15 with the currently known AKI score system.

## Conclusion

In summary, GDF-15 is an independent risk factor for the presence of AKI in AMI patients. GDF-15 and AKI predicate the short-term prognosis of AMI patients. Preoperative detection of GDF-15 helps AMI patients to carry out AKI risk stratification, thus early identification of high-risk patients and early preventive procedure might be executed.

## Supporting information

S1 Table Stratification analyses by age and serum creatinine in matched cohort. Presented are stratification analyses by age and serum creatinine in a matched cohort with 212 patients.
(PDF)

S2 Table Propensity score regression adjustment in overall cohort. PS = propensity score; propensity score to a 4-digit stratified assignment (1, 2, 3, and 4). Model1: unadjusted; Model2: adjusted for age, gender; Model 3: adjusted for age ≥ 70, gender, smoking, alcohol intake, hypertension, diabetes mellitus, anemia, Killip class ≥ 3, eGFR < 60ml/min/1.73m^2^, use of isotonic contrast agents, LAD, LCX, RCA, PCI, WBC, Neutrophil ratio, serum creatinine, Uric acid, HDL-C, HbA1c ≥ 7.0%, contrast volume > 150 ml, contrast exposure time > 60 min.
(PDF)

S3 Table Propensity score regression adjustment in matched cohort. PS = propensity score. Propensity score to a 4-digit stratified assignment (1, 2, 3, and 4). Model1: unadjusted; Model2: adjusted for age, gender; Model 3: adjusted for serum creatinine, eGFR, left anterior descending (LAD), use of isotonic contrast agents and use of diuretics.
(PDF)
