# Supplementary material for: Growth differentiation factor-15 levels and the risk of contrast induced acute kidney injury in acute myocardial infarction patients treated invasively: A propensity-score match analysis
Source: PLoS One. 2018 Mar 12;13(3):e0194152. doi: 10.1371/journal.pone.0194152 (PMC5846798; doi:10.1371/journal.pone.0194152)
Supplement: S1 Table — Presented are stratification analyses by age and serum creatinine in a matched cohort with 212 patients. (PDF) [file pone.0194152.s001.pdf]

S1 TABLE Stratification analyses by age and serum creatinine in matched cohort

	n	β	Wald chi-square	P Value	OR （95%CI）
GDF-15 Per 1000 ng/L (Age>50 years)	191	1.085	6.796	0.009	2.960(1.309-6.694)
GDF-15 Per 1000 ng/L (Age≤50 years)	21	-0.045	0.001	0.971	0.956(0.082-11.155)
GDF-15 Per 1000ng/L (Cr>60 μmol/L)	194	0.959	5.515	0.019	2.609(1.172-5.5810)
GDF-15 Per 1000 ng/L (Cr≤60 μmol/L)	18	0.782	0.320	0.572	2.186(0.146-32.817)

Presented are stratification analyses by age and serum creatinine in a matched cohort with 212 patients.
