# Supplementary material for: Growth differentiation factor-15 levels and the risk of contrast induced acute kidney injury in acute myocardial infarction patients treated invasively: A propensity-score match analysis
Source: PLoS One. 2018 Mar 12;13(3):e0194152. doi: 10.1371/journal.pone.0194152 (PMC5846798; doi:10.1371/journal.pone.0194152)
Supplement: S2 Table — PS = propensity score; propensity score to a 4-digit stratified assignment (1, 2, 3, and 4). Model1: unadjusted; Model2: adjusted for age, gender; Model 3: adjusted for age ≥ 70, gender, smoking, alcohol intake, hypertension, diabetes mellitus, anemia, Killip class ≥ 3, eGFR < 60ml/min/1.73m2, use of isotonic contrast agents, LAD, LCX, RCA, PCI, WBC, Neutrophil ratio, serum creatinine, Uric acid, HDL-C, HbA1c ≥ 7.0%, contrast volume > 150 ml, contrast exposure time > 60 min. (PDF) [file pone.0194152.s002.pdf]

**S2 TABLE Propensity score regression adjustment in overall cohort**

| | $\beta$ | Wald chi-square | P Value | OR （95%CI） |
|---|---|---|---|---|
| GDF-15 (Model1 plus PS) Per 1000 ng/L increase | 1.343 | 18.924 | <0.001 | 3.829(2.091-7.010) |
| GDF-15 (Model2 plus PS) Per 1000 ng/L increase | 1.350 | 19.074 | <0.001 | 3.856(2.104-7.067) |
| GDF-15 (Model3 plus PS) Per 1000 ng/L increase | 1.319 | 15.537 | <0.001 | 3.740(1.941-7.205) |

PS = propensity score; propensity score to a 4-digit stratified assignment (1, 2, 3, and 4). Model1: unadjusted; Model2: adjusted for age, gender; Model 3: adjusted for age ⩾ 70, gender, smoking, alcohol intake, hypertension, diabetes mellitus, anemia, Killip class ⩾ 3, eGFR < 60ml/min/1.73m$^2$, use of isotonic contrast agents, LAD, LCX, RCA, PCI, WBC, Neutrophil ratio, serum creatinine, Uric acid, HDL-C, HbA1c ⩾ 7.0%, contrast volume > 150 ml, contrast exposure time > 60 min.
