# Supplementary material for: Growth differentiation factor-15 levels and the risk of contrast induced acute kidney injury in acute myocardial infarction patients treated invasively: A propensity-score match analysis
Source: PLoS One. 2018 Mar 12;13(3):e0194152. doi: 10.1371/journal.pone.0194152 (PMC5846798; doi:10.1371/journal.pone.0194152)
Supplement: S3 Table — PS = propensity score. Propensity score to a 4-digit stratified assignment (1, 2, 3, and 4). Model1: unadjusted; Model2: adjusted for age, gender; Model 3: adjusted for serum creatinine, eGFR, left anterior descending (LAD), use of isotonic contrast agents and use of diuretics. (PDF) [file pone.0194152.s003.pdf]

S3 TABLE Propensity score regression adjustment in matched cohort

	β	Wald chi-square	P Value	OR （95%CI）
GDF-15 (Model1 plus PS) Per 1000 ng/L	0.859	4.715	0.030	2.360(1.087-5.123)
GDF-15 (Model2 plus PS) Per 1000 ng/L	0.884	4.923	0.027	2.420(1.109-5.282)
GDF-15 (Model3 plus PS) Per 1000 ng/L	0.873	4.018	0.045	2.395(1.020-5.626)

PS = propensity score. Propensity score to a 4-digit stratified assignment (1, 2, 3, and 4). Model1: unadjusted; Model2: adjusted for age, gender; Model 3: adjusted for serum creatinine, eGFR, left anterior descending (LAD), use of isotonic contrast agents and use of diuretics.
